# Delinquent Mortgages, Neglected Swimming Pools, and West Nile Virus, California 

**DOI:** 10.3201/eid1411.080719

**Published:** 2008-11

**Authors:** William K. Reisen, Richard M. Takahashi, Brian D. Carroll, Rob Quiring

**Affiliations:** University of California, Davis, California, USA (W.K. Reisen, B.D. Carroll); Kern Mosquito and Vector Control District, Bakersfield, California (R.M. Takahashi, R. Quiring)

**Keywords:** West Nile virus, California, anthropogenic factors, adjustable rate mortgages, swimming pools, dispatch

## Abstract

Adjustable rate mortgages and the downturn in the California housing market caused a 300% increase in notices of delinquency in Bakersfield, Kern County. This led to large numbers of neglected swimming pools, which were associated with a 276% increase in the number of human West Nile virus cases during the summer of 2007.

Although West Nile virus (WNV) (family *Flaviviridae*, genus *Flavivirus*) has remained epidemic in California since its arrival in 2003 (*1*), the cascade of events enabling local outbreaks remains poorly understood. WNV is amplified enzootically among several passeriform bird species within concurrent rural and urban cycles and is tangentially transmitted to humans by several *Culex* mosquito species (*2*). *Culex tarsalis* is the primary rural vector whose abundance relies on the availability of surface water created by precipitation and agricultural irrigation, whereas members of the *Cx*. *pipiens* complex are urban vectors whose abundance is dependent on underground drainage systems, wastewater, or anthropogenic peridomestic sources (*3*). Surveillance data useful in tracking WNV risk include temperature anomalies, mosquito abundance and infection rate trends, sentinel chicken seroconversions, dead bird reports and necropsy results, and the numbers of reported equine and human cases. Each of these factors are assigned a risk score, averaged, and ranked in terms of overall risk from 1 (normal season) to 5 (epidemic conditions) (*4*).

## The Study

An outbreak with 140 laboratory-confirmed human cases of WNV was centered in the Bakersfield area of Kern County, California, during 2007 (incidence = 17.5/100,000 population). This case cluster formed the WNV epicenter for California during 2007, was the largest mosquito-borne encephalitis virus outbreak in Kern County since the 1952 epidemic of western equine encephalitis virus (*5*) and represented a 205%–280% increase in the numbers of confirmed WNV cases observed since 2004 (*6*). The 2007 outbreak was unanticipated on the basis of surveillance data. Winter and spring weather was exceptionally dry (40% of expected rainfall) and hot (mean March–June temperatures ranged from 0.5°C–2.0°C above 30-year normal values). Rural *Cx*. *tarsalis* populations remained below 5-year averages (–32% to –76% of average during weeks 19–29) because of decreased rainfall, snow pack, and water allotments to agriculture. The Kern River, which flows through Bakersfield, remained mostly dry during spring and summer; key bird species decreased in abundance because of the drought (overall catch of free-ranging birds in 2007 was 31% of catch at the same traps during 2006) and the previous negative effect of WNV infection; and surviving birds in key species had high herd immunity to WNV (house finch WNV seroprevalence = 22%, n = 182; western scrub jay = 44%, n = 27) acquired during previous seasons.

Despite these findings, the infection incidence in *Cx*. *pipiens quinquefasciatus* increased rapidly to 18.5 females/1,000 mosquitoes in June 2007 at traps within Bakersfield, a month earlier than observed in previous summers (Appendix Figure 1). With reduced competition from house finches and predation on nestlings by western scrub jays, house sparrow populations increased dramatically. This expanding population was dominated by hatching year birds, had limited protective immunity (4.1%, n = 311), and comprised 23% of 124 WNV-positive dead birds reported by the public. Early season high risk of WNV infection in birds was followed closely by human cases, and this and several other case clusters of high incidence stimulated an emergency appropriation of $6.2 million by the Governor’s Office of the State of California to enhance surveillance and mosquito control.

**Figure 1:**
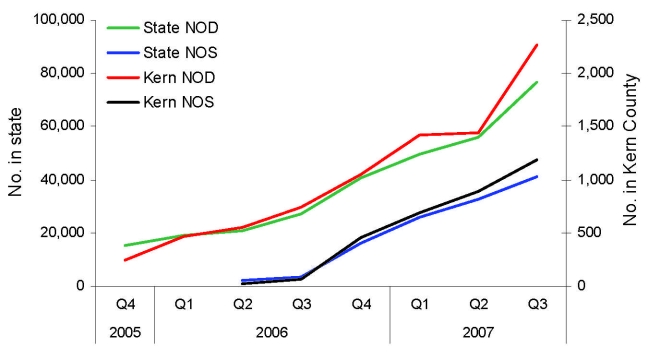
Notice of delinquency (NOD) and notice of sale (NOS) for homes in Kern County and California by quarter (Q) per year, 2005–2007.

**Figure 2:**
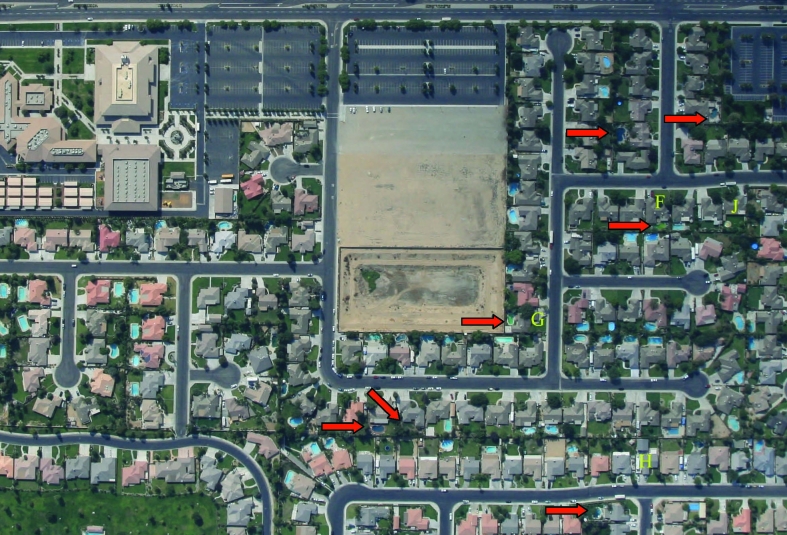
Aerial photograph of a representative Bakersfield, California, neighborhood taken during August 2007. Red arrows indicate neglected or green swimming pools. Letters (F, G, H, J) are photographic reference points.

Careful examination of service requests for mosquito control made to the Kern Mosquito and Vector Control District (KMVCD) and an aerial survey of Bakersfield showed an extensive number of green or neglected pools, most of which were producing mosquitoes. The likely reasons for neglected pools are the adjustable rate mortgage and associated housing crises in Kern County and throughout California, which have led to increased house sales, notices of delinquency of payment, declarations of bankruptcy and home abandonment. Kern County was especially affected (Figure 1), with a 300% increase in notice of delinquency in the spring quarter of 2007 compared with that of 2006. Associated with home abandonment was the expanding number of neglected swimming pools, Jacuzzis (hot tubs), and ornamental ponds. As chemicals deteriorated, invasive algal blooms created green swimming pools that were exploited rapidly by urban mosquitoes, thereby establishing a myriad of larval habitats within suburban neighborhoods that were difficult to locate from the ground. These pools frequently were located within new housing tracts and not confined to old neighborhoods. An aerial photograph of a representative Bakersfield neighborhood shows the extent of the problem, with 17% of the visible 42 pools and Jacuzzis appearing green and likely producing mosquitoes (Figure 2). The extent of this problem also was indicated by the marked increase in the number of pools that required treatment by the KMVCD (Appendix Figure 2). The increase in August 2007 followed an aerial survey of Bakersfield that enabled identification of previously unknown problem pools.

By law, all swimming pools or properties with pools have to be surrounded by 2-m high fencing and gates that must be locked when the homeowner is not present. These locked fences provided a formidable obstacle for mosquito control personnel to overcome for surveillance and treatment. Public awareness of this problem has been enhanced by education programs and media information, and local residents have begun to notify the KMVCD and other local agencies about neglected pool problems. Alarmingly, during 2008, many of these unmaintained pools previously positive for *Cx*. *p*. *quinquefasciatus* were now occupied by *Cx*. *tarsalis*, a more competent vector of WNV than *Cx*. *p*. *quinquefasciatus* (*7**,**8*). Collections of immature mosquitoes from 31 neglected pools taken during February–August 2008 produced 8,978 emerging adults, of which 59% were *Cx*. *tarsalis* and 41% were *Cx*. *p*. *quinquefasciatus*. Ongoing surveillance continues to monitor the extent of this problem in Kern County and throughout California and its affect on the ongoing WNV epidemic.

## Conclusions

Anthropogenic landscape change historically has facilitated outbreaks of pathogens amplified by peridomestic vectors such as *Cx*. *pipiens* complex mosquitoes and associated commensals such as house sparrows. The recent widespread downturn in the housing market and increase in adjustable rate mortgages have combined to force a dramatic increase in home foreclosures and abandoned homes and produced urban landscapes dotted with an expanded number of new mosquito habitats. These new larval habitats may have contributed to the unexpected early season increase in WNV cases in Bakersfield during 2007 and subsequently have enabled invasion of urban areas by the highly competent rural vector *Cx*. *tarsalis*. These factors can increase the spectrum of competent avian hosts, the efficiency of enzootic amplification, and the risk for urban epidemics.

## Supplementary Material

Appendix Figure 1 Monthly West Nile virus infection incidence for Culex spp. mosquitoes collected within the Kern Mosquito and Vector Control District in the Bakersfield, California, area during 2004-2007. An unexpected early season increase in Cx. p. quinquefasciatus infection rate (arrow) occurred during June 2007.

Appendix Figure 2 Number of swimming pools treated by mosquito control personnel per month in Bakersfield, California, 2005-2007. KMVCD, Kern Mosquito and Vector Control District.
