# Supplementary figures and images for: Delinquent Mortgages, Neglected Swimming Pools, and West Nile Virus, California
Source: Emerg Infect Dis. 2008 Nov;14(11):1747–9. doi: 10.3201/eid1411.080719 (PMC2630753; doi:10.3201/eid1411.080719)

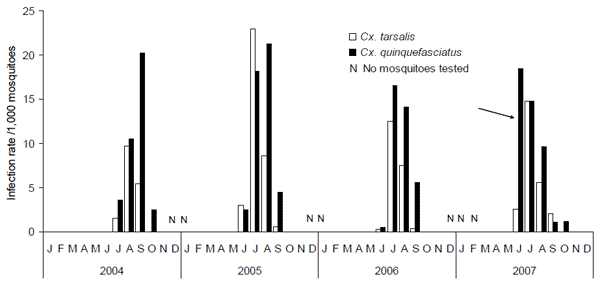

Supplement: Appendix Figure 1 — Monthly West Nile virus infection incidence for Culex spp. mosquitoes collected within the Kern Mosquito and Vector Control District in the Bakersfield, California, area during 2004-2007. An unexpected early season increase in Cx. p. quinquefasciatus infection rate (arrow) occurred during June 2007. [file 08-0719_app1-s1.gif]

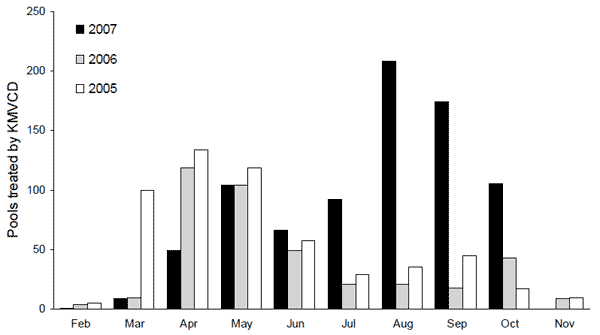

Supplement: Appendix Figure 2 — Number of swimming pools treated by mosquito control personnel per month in Bakersfield, California, 2005-2007. KMVCD, Kern Mosquito and Vector Control District. [file 08-0719_app2-s2.gif]
